# Current Practice in Bicistronic IRES Reporter Use: A Systematic Review

**DOI:** 10.3390/ijms22105193

**Published:** 2021-05-14

**Authors:** Guus Gijsbertus Hubert van den Akker, Federico Zacchini, Bas Adrianus Catharina Housmans, Laura van der Vloet, Marjolein Maria Johanna Caron, Lorenzo Montanaro, Tim Johannes Maria Welting

**Affiliations:** 1 Department of Orthopedic Surgery, Maastricht University, Medical Center+, 6229 ER Maastricht, The Netherlands; g.vandenakker@maastrichtuniversity.nl (G.G.H.v.d.A.); b.housmans@maastrichtuniversity.nl (B.A.C.H.); l.vandervloet@student.maastrichtuniversity.nl (L.v.d.V.); marjolein.caron@maastrichtuniversity.nl (M.M.J.C.); 2 Department of Experimental, Diagnostic and Specialty Medicine, Bologna University, I-40138 Bologna, Italy; federico.zacchini3@unibo.it (F.Z.); lorenzo.montanaro@unibo.it (L.M.); 3 Centro di Ricerca Biomedica Applicata—CRBA, Bologna University, Policlinico di Sant’Orsola, I-40138 Bologna, Italy; 4 Programma Dipartimentale in Medicina di Laboratorio, IRCCS Azienda Ospedaliero-Universitaria di Bologna, Via Albertoni 15, I-40138 Bologna, Italy

**Keywords:** bicistronic reporter, dicistronic reporter, mRNA translation, ribosome, IRES, systematic review

## Abstract

Bicistronic reporter assays have been instrumental for transgene expression, understanding of internal ribosomal entry site (IRES) translation, and identification of novel cap-independent translational elements (CITE). We observed a large methodological variability in the use of bicistronic reporter assays and data presentation or normalization procedures. Therefore, we systematically searched the literature for bicistronic IRES reporter studies and analyzed methodological details, data visualization, and normalization procedures. Two hundred fifty-seven publications were identified using our search strategy (published 1994–2020). Experimental studies on eukaryotic adherent cell systems and the cell-free translation assay were included for further analysis. We evaluated the following methodological details for 176 full text articles: the bicistronic reporter design, the cell line or type, transfection methods, and time point of analyses post-transfection. For the cell-free translation assay, we focused on methods of in vitro transcription, type of translation lysate, and incubation times and assay temperature. Data can be presented in multiple ways: raw data from individual cistrons, a ratio of the two, or fold changes thereof. In addition, many different control experiments have been suggested when studying IRES-mediated translation. In addition, many different normalization and control experiments have been suggested when studying IRES-mediated translation. Therefore, we also categorized and summarized their use. Our unbiased analyses provide a representative overview of bicistronic IRES reporter use. We identified parameters that were reported inconsistently or incompletely, which could hamper data reproduction and interpretation. On the basis of our analyses, we encourage adhering to a number of practices that should improve transparency of bicistronic reporter data presentation and improve methodological descriptions to facilitate data replication.

## 1. Introduction

Reporter gene assays are widely used to study cellular signaling, gene regulation, and mRNA translation. Reporter constructs, in their basic form, consist of a promoter of interest that controls the transcription of a reporter gene, thus allowing a quantitative reading of the transcriptional and translational activity. A plethora of reporter genes is available, ranging from purely enzymatic, luminescent, to fluorescent [1]. Each has its own advantages and limitations. For example, (radio)enzymatic assays, such as chloramphenicol acetyltransferase (CAT) or β-galactosidase (βGal) were used initially [2]. Later, luminescent proteins, such as Firefly (Fluc) or Renilla luciferase (Rluc) and their optimized reaction conditions led to the development of faster read-out [3]. More recently, fluorescent proteins, such as green fluorescent protein (GFP; [4]) have allowed for the sorting of specific living cells and other in vivo applications [5].

Most reporter assays utilize transient transfection of a cell line. Variation in transfection efficiency or cell number between experimental conditions or replicates can lead to misinterpretation of the results or high variation [6]. Therefore, it has become standard procedure to co-transfect a second constitutively active reporter plasmid to allow correction for such effects. Stable reporter lines have recently gained popularity, for example, in high-throughput screening procedures [7]. The generation of a stable reporter line requires a selectable marker to remove cells that did not incorporate the reporter. This can be achieved by utilizing antibiotic resistance genes or a fluorescent protein for sorting with a separate promoter or in a bicistronic configuration. In addition, some applications demand the possibility of utilizing two reporter genes (e.g., luminescent and fluorescent) at the same time, under the control of a single promoter. This can be achieved with an internal ribosome entry site (IRES), usually from the encephalomyocarditis virus (EMCV), or StopGo sequences, e.g., P2A from porcine teschovirus [8,9]. IRES mediated translation is a distinct mode of protein translation that was first discovered in viruses [10]. The IRES sequence can directly recruit the ribosome or through IRES transacting factors, which is independent of cap-mediated translation initiation, and may not require canonical initiation factors and ribosomal scanning [11]. A database with well-established IRES sequences currently contains >63 viral and >115 cellular IRESs [12].

In the past three decades, our understanding of viral IRES elements has improved significantly [11], and crucial determinants of IRES activity have been uncovered [13]. The use of bicistronic reporters has been instrumental to advance the field of IRES identification, their sequence variation, co-factor identification, and regulation. However, the study of bicistronic mRNA translation is not without potential pitfalls. In a cellular system using plasmid DNA transfections, cryptic splicing and cryptic promoter activity can take place [14]. Control experiments have been designed to rule out such confounding effects. For example, cryptic promoter activity can be evaluated by generating promoterless control plasmids, while cryptic splicing can be assessed with RT-qPCR, Northern blotting, or 5′RACE of reporter mRNA. An even more elegant approach is represented by siRNA targeting of one cistron in combination with RT-PCR [15,16]. Finally, read-through variation between evaluated control and test sequences can distort the results, which was recently shown to be avoidable using 2A StopGo sequences [17]. These 2A-sequences induce a premature stop of protein translation after a specific glycine and re-initiate formation of a new downstream polypeptide encoded at the next codon of the same mRNA. An alternative to plasmid DNA is the transfection of mRNA transcribed outside the cell, which allows for an integrity check by gel electrophoresis. This can also be combined with a cell-free translation assay [18]. A cellular lysate endowed with a high protein synthesis activity, often derived from a rabbit reticulocyte lysate (RRL), is combined with the mRNA in a reaction tube. The addition of specific co-factors to the system or depleting the lysate from endogenous ribosomes and replacing it with isolated ribosomes from sources of interest allows for in-depth ribosome activity studies [18]. Ribosomes can occasionally skip a stop codon, alter reading frames, or re-initiate translation [19]. Dedicated bicistronic reporter assays and analysis methods were developed to quantify such ribosome fidelity aspects [20].

The identification of eukaryotic IRESs in genes crucially involved in stress and apoptosis signaling (e.g., P53 [21]) has led to debates about limitations of bicistronic reporter assays and the definition of an IRES. With the advent of high throughput sequencing coupled to custom libraries, many new 5′ and 3′ UTR sequence elements (cap-independent translational elements, CITE) have been identified that alter cap-independent translation [22,23,24]. In addition, it has become apparent that the ribosome itself is not a static player in protein synthesis [25]. The protein composition or ribosomal RNA (rRNA) post-transcriptional modifications (PTM) affect its function [26,27]. Novel techniques to quantify small changes in rRNA PTM open up new possibilities for epitranscriptomics [28,29], which are expected to become increasingly complex when more types of RNA PTMs can be accurately quantified [30,31]. New insights in ribosome heterogeneity in stem cell differentiation [32], human diseases related to single gene defects (ribosomopathies [33]), or complex multifactorial diseases such as cancer [34] underline the importance of robust reporter assays to interrogate ribosome function. As such, the ribosome is now considered a drugable target for human disease [35,36].

In this review, we aimed to provide a focused and representative example of bicistronic IRES reporter use. For this purpose, we performed a systematic literature review to provide an unbiased overview of current practice in methodology, control experiments, and data reporting. Important inclusion criteria for further analysis were experimental studies using an adherent eukaryotic cell system or cell-free translation. We focused on technical and methodological aspects of experiments, as well as data visualization, control experiments, and normalization. Finally, we provide some basic recommendations for the use of bicistronic reporter assays that should benefit all users of bicistronic reporters. This is expected to contribute to better reproducibility of data and facilitate comparison of results between studies.

## 2. Results

**Figure 1:**
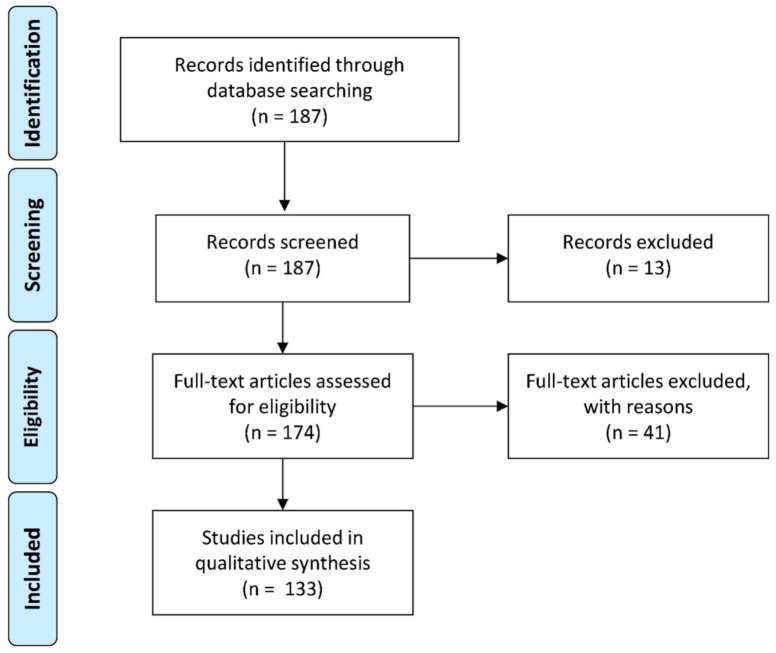
PRISMA flow diagram of eligible studies for this systematic review. Two hundred and fifty-seven studies were identified and screened. Twenty-eight studies were excluded after screening the title and abstract. Fifty-three additional studies were excluded on the basis of violation of inclusion criteria. A total of 176 full text articles were included in this study. The identity of all 257 articles and the 176 included studies can be found in Table S1.

**Figure 2:**
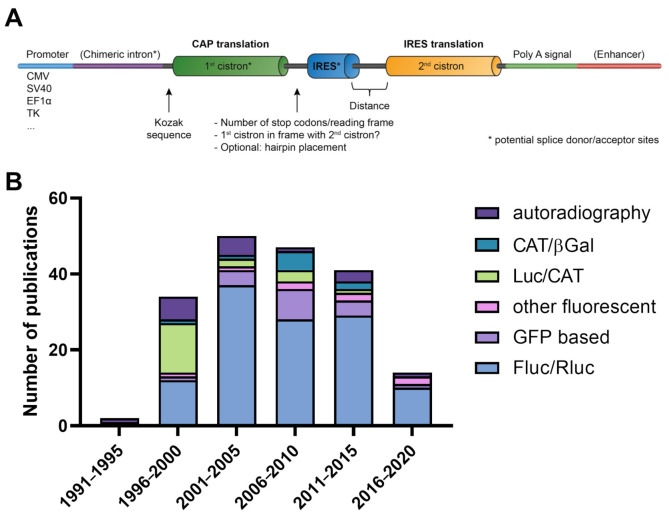
Bicistronic reporter design considerations and reporter gene use over time. (**A**) Relevant reporter design parameters are depicted. The choice of promoter and use of a chimeric intron and/or enhancer alters the mRNA expression levels, stability, and translation efficiency. The reporter genes themselves, as well as their positioning, can influence the observed IRES activity. The exact IRES sequence, for example EMCV sequence variants, can also have large effects. Potential read-through can be inhibited with (multiple) stop codons behind the first cistron (in all three reading frames) or inclusion of an inhibitory hairpin (second arrow). The first cistron always contains a Kozak sequence, but in some specific plasmid backbones, we identified a second Kozak sequence at the second cistron. A common criticism on the bicistronic reporter assay is the possibility of cryptic splicing due to specific reporter element (*) or cryptic promoter activity of the IRES sequence that can lead to false positive results. The former can be evaluated with Northern blot, RT-PCR, or 5′-RACE and ruled out with a monocistronic reporters or a cell-free translation system; the latter can be ruled out by using promotorless control constructs or a cell-free translation system. Cryptic promoter activity can be reduced with SPA sites, although this can lead new cryptic splicing events. (**B**) Reporter gene configuration was summarized by allocation to six functional groups in five-year bins. The first publication was from 1994, which explained the low number of publications in the first bin. βGal = β-galactosidase, CAT = chloramphenicol acetyl transferase, Luc = any luminescent protein, GFP = green fluorescent protein, Fluc = Firefly luciferase, Rluc = Renilla luciferase.

We selected 176 studies for evaluation of reporter system methodology, data representation, and normalization (Figure 1, Table 1 and Table S1, [37,38,39,40,41,42,43,44,45,46,47,48,49,50,51,52,53,54,55,56,57,58,59,60,61,62,63,64,65,66,67,68,69,70,71,72,73,74,75,76,77,78,79,80,81,82,83,84,85,86,87,88,89,90,91,92,93,94,95,96,97,98,99,100,101,102,103,104,105,106,107,108,109,110,111,112,113,114,115,116,117,118,119,120,121,122,123,124,125,126,127,128,129,130,131,132,133,134,135,136,137,138,139,140,141,142,143,144,145,146,147,148,149,150,151,152,153,154,155,156,157,158,159,160,161,162,163,164,165,166,167,168,169,170,171,172,173,174,175,176,177,178,179,180,181,182,183,184,185,186,187,188,189,190,191,192,193,194,195,196,197,198,199,200,201,202,203,204,205,206,207,208,209,210,211,212]). From these studies, 41 (23%) utilized a cell-free translation system. Relevant design parameters of a bicistronic reporter construct are summarized in Figure 2A. First, we evaluated the different types of reporter genes used over time (Figure 2B). In total, we identified 45 different reporter configurations. From 1994 until 2000, reporters based on luciferase and CAT were the most frequently used. The Fluc/Rluc luciferase system was by far the most popular from 2000 until the present. The use of autoradiography for cell-free translation assays was prevalent between 1994 and 2005. Fluorescent reporters acquired a modest but stable popularity from 2000 onwards.

### 2.1. Critical Determinants of Bicistronic Reporter Plasmid Design and IRES Activity

**Figure 3:**
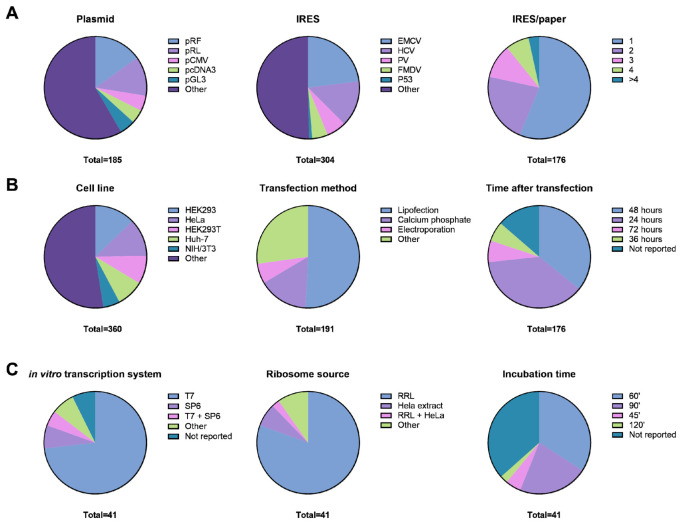
An overview of extracted information from included studies. (**A**) Plasmid, IRES, and the number of IRESs/publication were quantified and depicted in pie charts. (**B**) Cellular translation assay parameters. Transfection methods were combined in four categories for easier visualization. Time of transfection refers to the measurement time point of the reporter post-transfection. (**C**) Cell-free translation assay parameters. Note: total numbers do not always match the number of included studies due to the use of multiple parameters in one study.

Details on plasmid backbones were difficult to differentiate since the origin of the bicistronic reporter plasmids was not always traceable. In addition, modifications of various vectors (e.g., psiCHECKv2) to generate a bicistronic Fluc/Rluc reporter were often named pRF. As such, there is a large variety in the employed vector backbones (111 unique names) with limited traceability (Figure 3A, left panel). Aside from the CMV promoter, the SV40, HSV TK, PGK, and EIF1α promoters are often used to drive transcription of the bicistronic reporter, although we did not quantify the frequency of their use. Interestingly, a direct comparison of the same bicistronic EMCV IRES construct with either the CMV or PGK promoter exhibited a similar cap-mediated translation but a higher IRES-mediated translation with the PGK promoter [135]. A second study demonstrated higher IgG production with the EIF1α promoter when compared to CMV in a bicistronic vector, but this difference disappeared when swapping the cistron position [126]. The positioning of Fluc, Rluc, and CAT cistrons is known to affect IRES activity [73]. In terms of reporter types, the XIAP 5′ UTR was evaluated side by side in a Fluc/Rluc and a CAT/βGal system [109]. It was concluded that the CAT/βGal system appeared to be more reliable. The CMV promoter and intron A are known to contain a splice donor site [213]. The combination with an acceptor splice site within the coding sequence of Rluc and/or the 5′ UTR under investigation can lead to aberrant splicing and misinterpretation of cap-independent translational activity. Of note, it was shown that the bacterial pUC and pMB1 origin of replication sequences induced cryptic splicing and promoter activity in pGL3/pGL4 plasmids that can lead to false positive IRES activity [169]. Transcriptional pause sites (SPA) were introduced to counteract cryptic promoter activity, but this did not abrogate the cryptic splicing effects. The 3′ SV40 translational enhancer, which is present in pGL3-based Rluc/Fluc reporter constructs, was shown to mediate second cistron translation by cryptic promoter activity [114]. Transfection of in vitro-transcribed mRNAs from the same plasmid showed that cellular IRES translation (HIF-1α, VEGF, C-MYC, XIAP, VEGFR-1, or Egr-1) was lower than suggested by plasmid DNA transfection [114,214]. Therefore, it has been hypothesized that a nuclear event is required for efficient cellular IRES translation [214,215]. For example, HNRNPM and NONO were implicated in FGF1 IRES activity [216]. Another parameter that was evaluated in the literature is the number of nucleotides that are used as a spacer between an IRES element and the second cistron. An optimum of ≈30 nucleotides spacer sequence was established for the HTLV-1 and poliovirus IRESs [50]. In a similar study, increased spacer length (43–89 nt) decreased FMDV IRES activity [45]. In addition, the presence of 1–5 alternative start codons (in or out of frame) and the potential of hairpin formation in the spacer sequence reduced IRES activity. The ideal flanking sequence of the translation start codon was evaluated for the FMDV IRES and strongly affected eGFP protein expression [204]. Studies reporting on direct comparison of dual luciferase with dual fluorescence reporters were rare. At least for multiple HCV IRES sequence mutants both reporter configurations provided identical results in HEK293 and Huh-7 cells [164]. In the same study, stimulation of cells with IFNγ for 36 h demonstrated a higher sensitivity of the dual luciferase system in comparison to the fluorescent reporter. Two rare reporter gene configurations were used to enhance the signal from the first or second cistron. A 9 nt sequence derived from the Gtx 5′ UTR, which is complementary to a region of 18S rRNA, could strongly enhance second cistron translation [57]. Ten copies of this 9 nt sequence without spacers was 10-fold more active in a bicistronic Luc/CAT reporter than the EMCV IRES. In a different study, the signal of the reporter gene (Rluc or eGFP) was amplified by a positive feedback loop utilizing IRES-mediated translation of the yeast activator Gal4 and herpes simplex virus VP16 fusion protein (Gal4/VP16) that acts as a transcription factor [217] and Gal4 upstream activation sequences [113]. Next, we evaluated the employed IRESs. The total number of unique IRESs or 5′ UTRs in 176 studies was 116. Around one-quarter of the studies (23%) included the EMCV IRES, closely followed by the HCV IRES (14%; Figure 3A, middle panel). The first eukaryotic IRES in this list was P53 (1.0%). Although the majority of publications used a single IRES (56%), 40% of the publications used 2 to4 different IRESs, which excluded control constructs (Figure 3A, right panel). In conclusion, there are many important design parameters for a bicistronic reporter that affect its expression level. Because IRES bicistronic reporters have both been used and studied intensely, there is a large body of knowledge with various reporter gene configurations.

### 2.2. The Most Commonly Used Cell Lines and Cell Type-Specific IRES Activity

Cell lines were transiently transfected with reporter plasmids in nearly all studies, and few studies performed mRNA transfections. We focused on the following experimental details in these publications, amongst others: cell line(s), transfection reagent, and timing of reporter measurements (Table 1). The number of different cell lines [163] used in the analyzed publications was larger than the number of IRESs or 5′ UTRs (Figure 3B, left panel). Ninety-two studies used more than one cell line. The most popular cell lines were HEK293 (13%), HeLa (12%), HEK293T (9%), Huh-7 (8.6%) cells, and NIH/3T3 (5.3%), presumably because some of these cell lines are known to be easy to transfect. The high frequency of Huh-7 cell line use is due to the extensive study of the hepatitis C virus in this liver cell line and therefore also the HCV IRES. Other well-known cell lines that are often employed were HEPH2 (4.7%), Cos7 (3.4%), and MCF7 (3.0%). Only six studies utilized primary cells, such as mouse embryonic stem cells, embryonic fibroblasts, skin fibroblasts, neurons, or bone marrow-derived stromal cells [58,64,75,76,161,163]. A couple of studies stood out due to multiple comparisons between five or more cell lines and three or more viral IRESs [131,137,149,150,161,171,198]. In these studies, a certain cell line had an intrinsically high or low IRES activity, independent of the actual IRES under investigation. A notable exception was a comparative study of the FGF2 and EMCV IRES in 14 different cell lines [58]. FGF2 IRES activity was reported to be variable between cell lines, while the EMCV IRES activity was constant. GAL-4 5′ UTR activity was very similar to the cMyc IRES in five cell-lines [131]. Variants of the HAV IRES showed higher activity in the hepatocytic cell lines HepG2, Huh-7, and HLE, which was not the case in three non-hepatocytic cell lines: HeLa, BSC-1, and CV-1 [157]. Finally, hTPH2–5′ UTR variants showed variable reporter expression in six different cell lines, which was shown to be caused by cryptic promotor activity [146]. In summary, many different cell lines have been investigated with bicistronic IRES reporters. Since the number of different IRESs and cell lines is high, there are many unique combinations between IRESs and cell lines in the literature. However, comparative studies seem to indicate that this is less troublesome for data generalization than expected.

### 2.3. Methods for Transgene Expression and mRNA Translation In Vivo

Plasmid DNA or mRNA can be transiently transfected using various methods. Calcium phosphate transfection has been used for the longest time [218]. However, not all cell lines can be efficiently transfected with this method. Therefore, alternatives have been developed, such as electroporation [219], lipofection [220], and polymers [221]. We identified 39 different transfection reagents, of which Fugene 6 (17%) was the most used, closely followed by Lipofectamine 2000 (16%) and calcium phosphate (16%). A multitude of variants of Lipofectamine 2000, including Lipofectamin (+) and Lipofectin made lipofection the most frequently used transfection method (51%; Figure 3B, middle panel). Calcium phosphate was frequently used as well at 16%, and electroporation was used in 6% of the studies. Protocol parameters can affect the signal of specific cistrons. For example, increasing amounts of transfected plasmid DNA (10 ng to 5 µg) increased the cap-mediated signal without affecting the EMCV IRES signal [52]. Thus, the amount of plasmid DNA and consequently the mRNA levels can influence the observed IRES activity ratio. The use of viral reporter systems was rare in our dataset [71,76,121,147,149,172,178,188]. In retro-, lenti-, or adeno-associated virus constructs, the IRES itself was used to drive translation of a transgene. In viral reporter studies, the bicistronic IRES design was often directly compared to other reporter configurations (monocistronic, 2A StopGo sequence, etc.), usually for flow cytometry applications. A dose of lentivirus equivalent to 100 ng p24/24 wells effectively provided the most optimal luciferase signal in four different cell lines [149]. In one study, a tetracyclin-inducible IRES–GFP construct was generated and it was reported that aberrant splicing, aberrant polyadenylation, or fusion proteins could be generated [172]. Importantly, these issues depended on specific first cistron coding sequences. Overall, transfection protocol-related methodology is often reported with “according to manufacturer’s recommendations”. Relatively few studies address the effect of transfection reagents or protocol variables on bicistronic reporter activity.

### 2.4. Temporal Effects of Bicistronic Gene Transcription and mRNA Translation In Vivo

An important aspect of bicistronic reporter use is the time after transfection that is used for the measurement. The build-up, stability, and degradation of the reporter plasmid DNA, mRNA, and proteins affect the measured signal. Translation and/or stability of a reporter protein differs between reporter configurations, e.g., fluorescent proteins are known to require a longer maturation time to allow folding when compared to luciferases [222]. The most frequently used measurement time point after transfection was 24 or 48 h post-transfection with 38% and 36%, respectively. Less prevalent was 72 and 36 h with 6.8% and 6.3%, respectively (Figure 3B, right panel). The timing of measurements after transfection of the reporter was not reported in 14% of the cases. Nineteen studies measured at multiple time points after transfection. From time series experiments, it was shown that for plasmid DNA, the optimal time point to detect differences lies between 36 and 48 h post-transfection [205]. For the EMCV and HRV IRES, maximum activity in a Fluc/Rluc configuration could already be achieved at 12 h post-transfection [115]. Importantly, the exact sequence of the EMCV IRES in the construct had a large effect on the signal. For mRNA transfection, the largest differences were reported after 24 h in a time series experiment [160]. Studies that investigated multiple times points were often primarily focused on the effect of an IRES on viral replication [132,187]. In a few studies, the effect of a stimulus on IRES activity was measured as a function of time. In a 4–12 h hypothermia time series (30 vs. 37 °C), a consistent upregulation of viral IRES activity was found within 4 h that peaked at 12 h (equivalent to 36 h post-transfection) [198]. This was similar to a recent study with the ACC1 5′ UTR, where tunicamycin and thapsigargin induced peak IRES activity at 12 and 3 h, respectively [207]. HCV IRES activity was induced by hydrogen peroxide within 4 h in a 1–8 h time series [118]. A differential effect of IFNα on HAV IRES activity after 36 h was demonstrated in Huh-7 cells (down) and C13-3 cells (up), without an effect on cell viability [110]. The regulation of IRES-mediated translation over longer time periods has been minimally investigated. One study demonstrated differential use of the *Runx2* IRES in MC3T3-E1 over 14 days of differentiation using a stable transfection [101]. This IRES activity partially controlled Runx2 isoform expression. An in vivo knock-in mouse of the Utrophin (Utrn) IRES with CAT and βGal cistrons was generated to demonstrate tissue-specific activity of this IRES in skeletal muscle [158]. A Fluc/Rluc reporter containing the Fgf2 IRES was knocked-in to demonstrate differential effects of hyperglycemia depending on tissue type [103]. In conclusion, few studies address temporal regulation of IRES activity. In some studies, the time point of the measurement was not reported, which can hinder replication of the study. However, time series experiments seem to indicate that an increase or decrease in IRES activity can be measured over a broad time period. The ideal time point for measurement may differ between IRESs, and it was previously recommended to routinely perform time series experiments [6].

### 2.5. The Use of the Cell-Free Translation System

The cell-free translation system was used to test IRES activity and in most cases for comparison with the in vivo system. In addition, it was used to exclude potential cryptic splicing or promoter activity [154,156] or to investigate the effect of reaction parameters on translational susceptibility [174,201]. This assay was used in 41 of the included studies (23% of total), of which 10 studies utilized monocistronic reporters. The type of bicistronic reporter was primarily the Fluc/Rluc type with 55%, followed by Luc/CAT (29%). The in vitro transcription system to generate mRNAs was predominantly T7 RNA polymerase-based (73%; Figure 3C, left panel), while the used cell-free system was usually a rabbit reticulocyte lysate (80%; Figure 3C, middle panel). The incubation times for protein translation varied from 15 to 120 min, where 60 min was the most frequent (34%), and this incubation period was not reported in 37% of the studies (Figure 3C, right panel). The reaction temperature was 30 °C in 54% of the studies and 37 °C in 7% of the studies, and not reported in 34% of the cases. RRL samples are almost exclusively incubated at 30 °C and not at the physiological body temperature of a rabbit (≥37 °C; [223]) due to the observation that 37 °C incubation led to a premature stop of protein synthesis [224]. Of note, this problem was not reported with HeLa lysates that are incubated at 37 °C [77,107,201,225]. Certain parameters that we intended to evaluate were highly variable and not consistently reported, such as co-factors in the translation reaction (cap analogue, etc.), mRNA amount (moles, mass, concentrations), and purification method of the mRNA (gel extraction, phenol/chloroform, spin columns, or LiCl precipitation). None of the included studies using cellular IRESs reported the addition of cellular extracts to the RRL system. This was previously done to overcome the poor translation efficiency of entero and rhinoviral IRESs [226] and might be crucial for translation initiation of cellular IRESs. Of note, the ability of polypyrimidine tract-binding proteins to act as IRES transacting factors was demonstrated by adding them directly to the RRL [152]. In one study, mRNA concentration effects were investigated using MDV IRES mRNAs and a control mRNA not containing the IRES [153]. A strong increase in IRES activity was observed between 0.4 and 1.6 µM mRNA. Autoradiographical detection of signal with ^35^S-methioine and SDS-PAGE was more often used than bioluminescence (57% vs. 43%), although the latter has become more frequently used over the past 15 years. Importantly, the validation of cellular bicistronic reporter activity with a cell-free translation assay can reveal false-positive IRES activity in the bicistronic reporter related to cryptic promotor activity [146]. However, not all experimental conditions are consistently reported.

### 2.6. Visualization of Bicistronic Reporter Data: Ratio vs. Separate Cistrons

**Figure 4:**
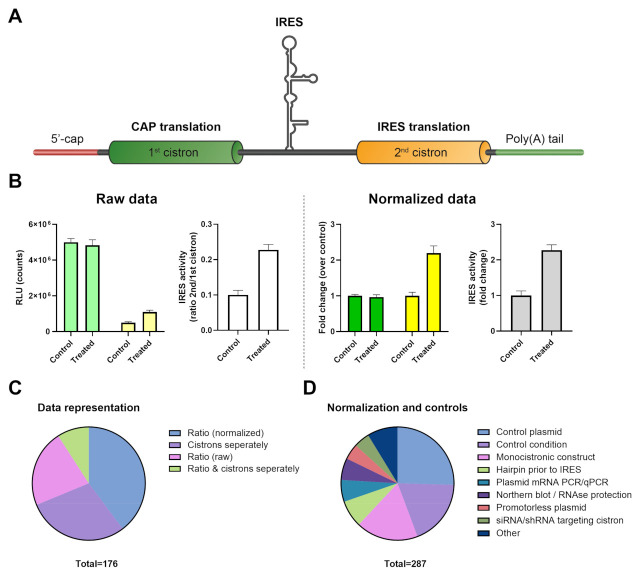
Data representation possibilities and overview. (**A**) Schematic of a bicistronic IRES reporter mRNA. (**B**) Visualization options for bicistronic data. Left: raw data from separate cistrons or the calculated ratio. Right: normalized cistrons separately and a normalized ratio. The latter is easier to interpret, however, relevant information is also lost about the measurement and relative signal differences. (**C**) Data representation in analyzed publications. (**D**) Normalization strategies and control use. Note that multiple normalization strategies or controls were combined in a large number of studies. The prevalence of the top eight normalization or control methods is depicted in the pie chart.

There are multiple ways to visualize and report bicistronic reporter data (Figure 4A). The activity ratio between the second and first cistron is often used for IRES bicistronic reporters, either as a ratio of the measurements or as a fold change (Figure 4B; white vs. gray bars).

Data from the individual cistrons can also be presented (Figure 4B: dark green/yellow bars). This provides additional information about what cistron was regulated when compared to reporting the ratio alone. The measured luminescence or fluorescence values can be provided in a table or graph to improve transparency (Figure 4B: light green/yellow bars). We analyzed the representation of the data to obtain an overview of the preferred strategies for data presentation (Figure 4C). The majority of studies showed the ratio between both cistrons, which was either the normalized ratio (40%) or actual ratio (22%). In 29% of the studies, only the activity of the individual cistrons was shown, which reflects the actual measurements. Only 9% of the studies showed both the ratio and individual cistron data. Of note, one-third of the articles showed the raw data (RLU for luciferase, CAT/βGal activity, or fluorescence intensity). Overall, reporting the ratio in combination with separate cistrons appears to be the most informative, preferably with a reference to the raw data in the figure legend or (supplementary) table when fold changes are graphed.

### 2.7. Normalization and Validation of Bicistronic Reporter Data

Normalization strategies are utilized to remove the potential influence of changes in cap-mediated translation, cell number, differences in transfection efficiency, or transcriptional activity, all of which can induce undesirable skewing of data in cellular assays. For example, cap-mediated Rluc signal can be normalized to *Rluc* mRNA copy number, Rluc plasmid copy number, β galactosidase activity, or total protein levels. Most of these parameters, which vary inherently in a cellular system, can be well-controlled in cell-free translation assays. Secondly, there are clear differences between the type of study and the controls they employ in the literature. IRES identification articles tend to use many different types of plasmid controls, for example, inclusion of hairpins between cistrons, monocistronic and promotorless variants, and mutations or truncations of the 5′ UTR/IRES. Articles that utilize an established IRES often lack these types of control plasmids, although an empty plasmid backbone can be used. A single control was used in 24% of the studies, the majority used 2–3 controls (63%), and ≥4 controls was less frequent (13%). The most frequently used type of control was an empty control plasmid and sometimes a highly active IRES for reference (25%; Figure 4D). In 19% of the cases, a control condition with the same IRES construct, for example unstimulated or non-treated cells, was used. Monocistronic constructs to validate findings were used in 18% of the cases. Inclusion of a hairpin prior to the IRES (7.7%), plasmid-derived mRNA analyses or Northern blot, and RNase protection assays (6.3% each) were also used quite often. Controls that were less frequently used are a promoterless control vector (4.5%), siRNA or shRNA targeting of cistrons (4.6%), cell viability test (MTT, XTT, and others; 2.4%), an indication of non-transfected cell background level or correction (2.1%), normalization to total protein levels in a lysate (1.4%), comparison of capped and uncapped in vitro-transcribed RNA (0.7%), a swap in the orientation of the IRES from 5′-3′ to 3′-5′ (1.4%), or plasmid DNA level correction (0.7%). In summary, a multitude of controls can be used to validate identification of a new IRES to prevent incorrect interpretation of the bicistronic reporter data. For established IRESs, normalization controls are still useful. The use of an empty control plasmid appeared to be a straightforward, frequently used, and informative control condition.

## 3. Discussion

In this systematic review, we characterized in an unbiased manner how the bicistronic reporter assay is used from a methodological perspective. We focused on IRES-mediated translation since this is the most frequently used, sensitive, and critical application for this type of reporter. A plethora of technical studies covering plasmid characteristics, sequence variation, and undesirable cryptic splicing or promoter activity has been conducted in the past three decades. New developments in the field have led to the discovery of a multitude of cap-independent translational elements [23] and new evidence for specialized ribosomes [227], which ultimately requires stringent validation using this type of reporter.

Although we analyzed a significant number of articles, it should be noted that we did not identify and include some well-known and highly cited articles in the IRES field [228,229,230,231]. This result could not be avoided, since analyzing all articles on IRESs would be an insurmountable task. Despite this limitation, we obtained a significant representation on the use and reporting of bicistronic IRES reporters. Ultimately, this should lead to more transparency and easier reproducibility of published findings. Naturally, many different studies were analyzed, with different research objectives, such as viral IRES mechanistic studies, cellular IRES identification studies, cellular IRES regulation studies, and studies where a bicistronic reporter with an IRES was used. However, this review should provide a comprehensive overview of the critical assay parameters that have been evaluated in a systematic manner for all potential users.

The most used bicistronic reporter configuration was the Fluc/Rluc reporter gene combination. This is likely related to the high sensitivity, linearity of the signal, similar maturation times, and proteolytic stability of these luciferases [232]. Although CAT/βGal reporters see limited use in recent times, their susceptibility to cryptic splicing was shown to be lower than Fluc/Rluc reporters [109]. Fluorescent reporters can be used for flow cytometric applications; however, reporter protein maturation times are substantially longer [222], and therefore sensitivity was demonstrated to be lower [164]. To our knowledge, newer luciferases, such as NanoLuc derived from *Oplophorus gracilirostris* [233], have not yet been used in bicistronic reporter configurations. Of note, for dual expression of GFP and RFP, it was shown that a 2A StopGo sequence showed better co-expression characteristics than the EMCV IRES [210]. This is likely related to the different mode of translation. Therefore, if the sole objective is to co-express two cistrons from one mRNA, 2A StopGo sequences should be seriously considered.

In general, it was challenging to deduce the exact reporter construct composition, due to extensive back referencing and limited availability of sequences and bicistronic IRES reporter constructs in literature and plasmid repositories. For example, the search terms used for this study only identified two Fluc/Rluc IRES reporter plasmids at the Addgene repository (#45642 and #18673), while we found over 111 unique plasmid names in the literature. Many research groups generated their own bicistronic Fluc/Rluc reporter that often received the same abbreviation (pRF), which adds to potential confusion. With current next-generation sequencing techniques, we recommend making plasmid backbones and IRES sequences available upon publication. A nice example is the development of a dual luciferase reporter backbone with flanking 2A StopGo sequences to prevent read through (Addgene #119760, [17]), although this vector has not yet been used to evaluate IRES mediated translation.

The EMCV IRES was the most used in the analyzed literature, presumably due to its high activity. However, we also found the use of a large number of different IRESs or 5′ UTRs in an even higher number of different cell lines. This implies that many findings have been obtained using a specific IRES or 5′ UTR in a specific cell line that have not been replicated by an independent group. As a result, there is limited data about cell line or cell type-specific activity of one IRES or 5′ UTR. This cell type specificity is highly relevant in the context of ribosome heterogeneity and cellular differentiation or disease-specific mRNA translation studies [32,34].

The most commonly used transfection method was lipofection, while stable reporter cell line use is relatively rare. The latter is presumably related to the continuous mRNA expression of such a stable transgene, where reporter protein turnover becomes a factor. An inducible system might be an elegant solution that could facilitate temporal regulation studies. Reporting of relevant transfection methodology is usually done, and it is recommended to at least specify the amount of transfected DNA/RNA, number of cells, confluency, and incubation times. In cell-free translation systems, the lack of reporting such parameters could be motivated by the fact that commercial assays and protocols are often used. Measurement time points post-transfection were usually between 24 and 48 h. For specific treatments (hypothermia, hydrogen peroxide, and IFNɑ), alterations in IRES translation were observed within 4 h post-stimulation, and peak inductions were observed at later time points up until 12 to 36 h. This is consistent with the notion that translational regulation is a potent mechanism to modify protein expression [234]. Transgenic knock-in mice provided evidence for cell type-specific activity of the Fgf2 IRES and the Utrn 5′ UTR [58,103,158]. Temporal regulation of eukaryotic IRES or 5′ UTR translation in the context of environmental changes is currently understudied since most publications have focused on identifying new IRESs or CITEs. In other study types, the EMCV IRES, which was shown to provide stable second cistron expression in many cell lines and cell types [58], was used to translate the second cistron. In cell-free translation systems, the most used lysate source for translation is the well-known RRL, despite the known problems associated with this system [6]. This is probably due to its wide diffusion, commercial availability, and the lack of valid commercial alternatives. However, the use of cell-free translation systems, in particular RRL, can lead to low translation efficiency in the study of some cellular IRESs that require a “nuclear experience” [214,215,235]. For these cellular IRESs, the availability of particular nuclear IRES transacting factors is thought to be required for their proper translation initiation. For this reason, RRL is sometimes supplemented with ribosomal salt-wash fractions from cell extract [236], but none of the included articles used these co-factors. The use of bicistronic reporters in cell-free assays is complex and only used by a specialized minority of researchers. Despite this, the considerations and problems encountered are in part similar to those of in vivo assays, although cryptic promoter activity or undesired splicing problems are avoided.

**Figure 5:**
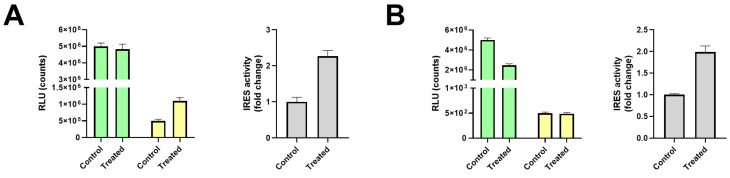
Potential misinterpretation of activity ratios without individual cistron data. Reporting of ratios alone can be misleading due to different underlying reasons for regulation. For example: (**A**) IRES ratio induction, caused by an increase of second cistron activity. (**B**) IRES ratio induction, due to a decrease of first cistron activity. Note that IRES activity in this hypothetical example is very low and might be close to the detection limit or background levels. Naturally, both first and second cistrons can also be induced or reduced, wherein slight differences can lead to IRES induction or repression. Additional normalization strategies can be used to correct for changes in cap translation (monocistronic βGal co-transfection), cell number, or protein content. Graphs were generated for illustration purposes using hypothetical data, on the basis of our own experience with the bicistronic reporter assay.

Data presentation of bicistronic reporters can be performed in multiple ways. In the pre-2000 era, we found a majority of articles that showed only data of individual cistrons. Presenting the ratio of both cistrons alone is a more modern development that is less transparent and might be misleading. In an ideal IRES study, cap-mediated translation would remain equal as a result of a treatment compared to control and the IRES-mediated translation would be induced (Figure 5A). However, a decrease in cap-mediated translation and equal IRES-mediated translation could result in a similar IRES activity ratio change (Figure 5B). This last example can also lead to misinterpretation if the observed IRES activity is close to background levels: IRES signal will remain the same, while cap signal is reduced, leading to the false-positive conclusion that IRES activity is increased. Cap and IRES activity can both be negatively affected by a treatment, such as apoptosis induction [237] or amino acid starvation [238]. Reporting the ratio in combination with presenting data from the separate cistrons appears to be the most informative way of presenting these kinds of data, preferably with a reference to the raw data and background levels when fold changes are graphed.

Normalization of bicistronic reporter data is not always performed, and this depends largely on the study purpose. For bicistronic reporter experiments where the activity of the element between the two cistrons is not under investigation, such normalization is often not used. In general, care should be taken to prevent misinterpretation related to transcriptional activity, cell viability, cryptic splicing, or cryptic promoter activity. The fact that such limitations have mostly been described within the IRES field using Fluc/Rluc reporters does not imply that these issues do not take place in other fields or other reporter configurations. For most cellular studies, it is feasible to normalize to cell number (DNA content), total protein content, or cell viability, and such normalization approaches would certainly strengthen data interpretation.

A control condition (vehicle or non-treated) is commonly used in studies on IRES translation regulation following a stimulus or change in environment. Negative control plasmids are often used for investigating new 5′ UTRs for potential CITE or IRES activity, and we consider this as an important control that could be more widely used. In addition, the use of a positive control, such as the EMCV IRES, would provide an elegant reference point. Additional hairpin structures 5′ of the IRES, sometimes in combination with a monocistronic reporter, are often used to strengthen the cap-independent translation data (cf. Figure 2A). It is unfortunate that the most often used negative control hairpin sequences (∆EMCV [239], P53 hairpin [240,241]) are challenging to trace down in the literature, although they might be well known in the field. Reporter mRNA levels or integrity are often verified in translational studies employing plasmid DNA, which provides additional indication for IRES or CITE activity. Northern blotting has not commonly been performed to address IRES reporter integrity since 2010, but (q)PCR-based techniques can be easily implemented.

In summary, on the basis of our literature review, we encourage researchers to adhere to the following recommendations for publication of bicistronic reporter data:

(1) Make plasmid backbones and IRES sequences available upon publication.
(2) Carefully describe transfection procedures and the timeline of the experiment.
(3) Report both the ratio and individual cistron data.
(4) Make raw data available, including background signals.
(5) Verify whether treatments affect cell number, total protein content, or cell viability.
(6) For cellular IRES translational studies, an empty control plasmid to define background signals; a promoterless control plasmid to assess cryptic promoter activity of the IRES; and verification of equal and intact mRNA levels by RT-qPCR and RT-PCR, respectively, are feasible and represent important controls.

More stringent controls have been recommended for the identification of eukaryotic IRESs (reviewed in [6,14]). These recommendations center on favoring mRNA transfections over plasmid DNA transfections and the generation of an extensive set of control mRNAs. These include an empty control (or inversed 5′ UTR), a proximal stable hairpin, and monocistronic variants with and without cap. In our literature search, we found only four articles that adhered to these recommendations [43,160,165,205]. In addition, it is known that eukaryotic IRESs or CITEs have strongly reduced activity in mRNA transfection experiments, which has been used to question their existence or to argue for the existence of a nuclear experience of the mRNA [14,242]. Bicistronic reporters were often combined with monocistronic variants and an empty control plasmid, but introduction of a hairpin and cap vs. uncapped comparisons were rare. Naturally, once novel CITE or IRESs are established, follow-up studies might not require the same set of controls. However, our recommendations 1–6 would still be applicable for slightly different IRES sequences and different plasmid backbones. Finally, this study also has broader relevance for the optimization of bicistronic reporter use to drive transgene expression.

## 4. Methods

### Search Strategy and Inclusion Criteria

We were interested in reported methodological details, data representation, normalization, and control experiments in articles on bicistronic reporters. Since IRESs are historically the most utilized and studied bicistronic elements, we focused on this in particular. Where possible we applied the PRISMA guidelines for a systematic review [243].

Pubmed, a repository of over 30 million citations to MEDLINE; life science journals; and online books were systematically searched with the keywords “IRES + bicistronic + reporter” and “IRES + dicistronic + reporter”. The number of identified articles was 257, and these were published between 1994 and 2020 (Table S1). The keywords “bicistronic + reporter” and “dicistronic + reporter” led to a large number of articles (571 studies, published between 1989 and 2020). It should be noted that we covered a significant portion of these articles (257/571) by including the IRES search term, while keeping the number of articles manageable. Although the term CITE has recently gained popularity, replacing IRES with CITE in the search terms yielded one article.

Review articles were excluded from analysis, since they lack new data and methodological procedures (Figure 1, screening). In addition, we focused on adherent eukaryotic cell systems, and therefore bacterial, fungal, and yeast studies were excluded (Figure 1, eligibility). Substantial lack of methodological details (such as transfection method and plasmid information) was also a reason for exclusion of the article for further analysis. Finally, the article should contain a bicistronic reporter construct with an IRES or 5′ UTR. Therefore, studies were excluded if they utilized an IRES merely to drive an antibiotic resistance gene for selection and not for reporter purposes. Due to strong methodological differences between in vivo and in vitro translation assays, we dedicated a separate paragraph to the cell-free translation system. Three independent researchers (G.G.H.v.d.A., F.Z., and L.v.d.V.) excluded studies on the basis of the inclusion criteria for cellular systems, while two other independent researchers checked this for the cell-free translation system (F.Z., L.M.).

The extracted parameters that we defined and to which assay (in vitro or in vivo translation) they apply are indicated in Table 1. An arbitrary selection was made for visualization in pie charts. Certain parameters (e.g., co-factors in in vitro translation) were so little reported that we refrained from reviewing them here.

## Figures and Tables

**Table 1:** Overview of information extracted from the 176 included studies.

Collected Information	In Vivo Translation	In Vitro Translation
PubMed ID, authors and title	Y	Y
Year of publication	Y	Y
The plasmid backbone name(s)	Y	Y
The bicistronic reporter gene configuration	Y	Y
Monocistronic reporter use (yes/no)	Y	Y
The name of the investigated IRES or 5′ UTR	Y	Y
The number of iress or 5′ UTRs studied in one article	Y	Y
The name of used cell line(s) or primary cell type	Y	NA
The number of cell lines used in one article	Y	NA
The name of the transfection reagent (if applicable)	Y	NA
Reported cell density (yes/no, and if yes, what was mentioned)	Y	NA
Time post-transfection/translation initiation of the measurement	Y	Y
Method of reporter protein detection	Y	Y
In vitro transcription system	NA	Y
mRNA isolation method	NA	Y
mRNA amount	NA	Y
Lysate type (source of ribosome preparation)	NA	Y
Temperature of reaction °C	NA	Y
Co-factors (when reported)	NA	Y
**Data presentation categories**	**In vivo translation**	**In vitro translation**
Ratio (raw)	Y	Y
Ratio (normalized)	Y	Y
Ratio and cistrons separately	Y	Y
Cistrons separately	Y	Y
Raw data available (RLU/RFU/CPM)	Y	Y
**Normalization and control categories**	**In vivo translation**	**In vitro translation**
Correction for empty reporter or IRES positive control	Y	Y
Correction for control condition (same reporter, non-treated cells)	Y	Y
Correction for total protein levels	Y	Y
Correction for reporter mRNA levels	Y	Y
Background of non-transfected cells shown	Y	NA
Background subtraction/correction	Y	NA

The table is divided in three parts: collected information, data presentation, and normalization/controls (indicated in bold). Y (yes) or not applicable (NA) indicates whether a parameter applies to in vivo or in vitro translation assays.

## Data Availability

Data is contained within the article or supplementary material.

## Supplementary Materials

The following are available online at https://www.mdpi.com/article/10.3390/ijms22105193/s1, Table S1: Overview of identified and included research articles.
